# A Molécula de Lesão Renal-1 está Associada à Nefropatia Induzida por Contraste em Pacientes Idosos com IAMSSST

**DOI:** 10.36660/abc.20200172

**Published:** 2021-06-08

**Authors:** Mustafa Ahmet Huyut

**Affiliations:** 1 Yeni Yuzyil University Faculdade de Medicina Departamento de Cardiologia Istambul Turquia Yeni Yuzyil University, Faculdade de Medicina, Departamento de Cardiologia, Istambul - Turquia

**Keywords:** Nefropatias/induzido quimicamente, Infarto do Miocárdio SST, Intervenção Coronária Percutânea

## Abstract

**Fundamento:**

A nefropatia induzida por contraste (NIC) está associada a um risco aumentado de eventos cardiovasculares adversos maiores (ECAM), e a associação entre NIC e mecanismos oxidativos está bem documentada.

**Objetivo:**

Este estudo visou avaliar a relação entre os níveis séricos da molécula de lesão renal-1 (KIM-1) e a NIC em pacientes idosos com infarto do miocárdio sem supradesnivelamento do segmento ST (IAMSSST).

**Métodos:**

O presente estudo incluiu um total de 758 pacientes com IAMSSST que foram submetidos a intervenção coronária percutânea (ICP); 15 desenvolveram NIC após a ICP e outros 104 constituíram o grupo controle, pareado por idade > 65 anos. Foram registrados os valores laboratoriais desde a linha de base até o período entre 48 e 72 horas e os achados clínicos. Os pacientes foram acompanhados durante um ano. Foram considerados significativos valores de p < 0,05.

**Resultados:**

A NIC foi observada em 12,60% dos pacientes. A KIM-1 sérica foi significativamente mais alta no grupo com NIC que no grupo sem NIC (14,02 [9,53 – 19,90] versus 5,41 [3,41 – 9,03], p < 0,001). O escore Mehran foi significativamente mais alto no grupo com NIC do que no grupo sem NIC (14 [5 – 22] versus 5 [2 – 7], p = 0,001). Os ECAM foram significativamente maiores no grupo com NIC do que no grupo sem NIC (7 [46,70%] versus 12 [11,50%], p = 0,001). A análise de regressão logística multivariada mostrou que o nível de KIM-1 basal (OR = 1,652, IC 95%: 1,20 – 2,27, p = 0,002) e o escore Mehran (OR = 1,457, IC 95%: 1,01 – 2,08, p = 0,039) foram preditores independentes da NIC em pacientes idosos com IAMSSST.

**Conclusão:**

A concentração sérica basal de KIM-1 e o escore de Mehran são preditores independentes de NIC em pacientes idosos com IAMSSST. Além disso, todas as causas de mortalidade, morte cardiovascular, reinfarto do miocárdio, acidente vascular cerebral e MACE foram significativamente maiores no grupo CIN no acompanhamento de um ano. (Arq Bras Cardiol. 2021; [online].ahead print, PP.0-0)

## Introdução

A nefropatia induzida por contraste (NIC) está associada a um aumento de morbimortalidade e de hospitalizações, devido à aplicação de meios de contraste (MC) intravenosos ou intra-arteriais durante os procedimentos vasculares diagnósticos ou terapêuticos.^1^ A incidência da NIC frequentemente varia de acordo com as populações estudadas e com as suas comorbidades relacionadas.^2^ Os mecanismos subjacentes da NIC incluem disfunção endotelial, inflamação, vasoconstrição, toxicidade das células tubulares, lesão de radicais livres, espécies reativas de oxigênio, estresse oxidativo, e ativação de neutrófilos e plaquetas, que causam a liberação de radicais livres de oxigênio, enzimas proteolíticas e mediadores pró-inflamatórios que podem causar dano tecidual e endotelial, particularmente em miócitos criticamente lesionados.^3,4^ O ácido úrico, a largura de distribuição dos glóbulos vermelhos, a proporção de plaquetas para linfócitos e a proporção de neutrófilos para linfócitos foram correlacionados com a NIC em estudos prévios.^5,6^ A molécula de lesão renal-1 (KIM-1) tem sido relacionada à ocorrência e à gravidade da lesão renal aguda e da doença renal crônica.^7^ A KIM-1 é uma proteína transmembrana do tipo 1, expressa de acordo com a lesão no túbulo proximal da membrana apical.^8^ A doença cardiovascular possui uma forte ligação com a lesão renal aguda e a doença renal crônica, e tem sido relatado que eventos cardiovasculares são associados à lesão renal aguda.^9^ A KIM-1 serve como um agente pró-inflamatório com funções de adesão celular.^7^ Na literatura, existem alguns estudos publicados sobre a relação entre a KIM-1^10,11^ e escores de Mehran^12,13^ no desenvolvimento da NIC, mas estudos prévios não mencionaram qual desses seria o melhor preditor. Adicionalmente, os estudos anteriores não fizeram uma comparação entre a KIM-1 e o escore de Mehran para predizer o desenvolvimento da NIC em pacientes idosos.

A nossa hipótese foi de que a expressão da KIM-1 é induzida em pacientes idosos com infarto do miocárdio sem supradesnivelamento do segmento ST (IAMSSST) e está relacionada à NIC devido à resposta pró-inflamatória e que o dano endotelial tubular proximal ocorre dessa forma. Ainda não foi abordada na literatura a associação entre os níveis de proteína de KIM-1 e a NIC em pacientes idosos com IAMSSST. Compreender quais vias biológicas e marcadores estão associados à NIC pode permitir o desenho de estudos futuros para explorar a ligação mecanicista entre essas vias e para avaliar a eficácia das intervenções projetadas para reduzir a carga das doenças cardiovasculares e da NIC nesses pacientes. Por isso, este estudo visou avaliar a relação entre os níveis séricos basais da proteína KIM-1 e a NIC em pacientes idosos com IAMSSST.

## Métodos

**Figura 1:**
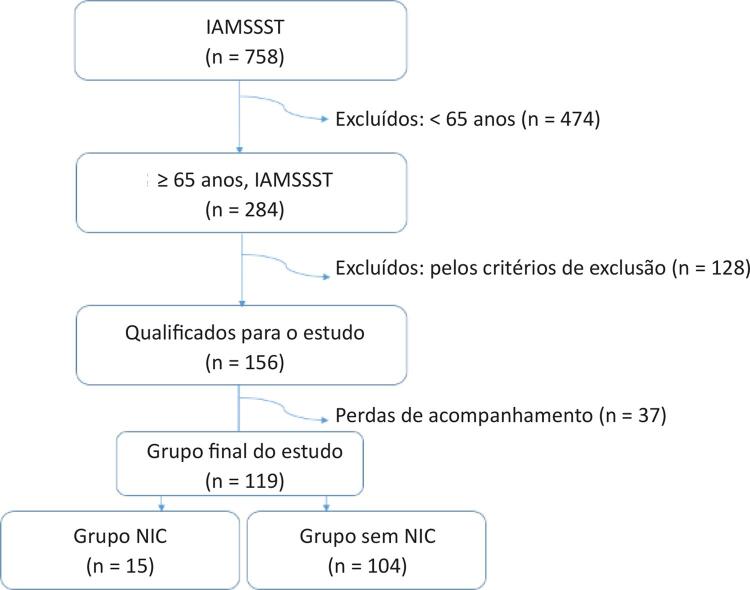
– Seleção dos grupos do estudo. IAMSSST: infarto do miocárdio sem supradesnivelamento do segmento ST; NIC: nefropatia induzida por contraste.

O presente estudo foi conduzido prospectivamente entre julho de 2016 e julho de 2018 no Hospital Universitário Bezmialem Vakif. Incluímos 758 pacientes que foram diagnosticados com IAMSSST e que foram submetidos a ICP precoce dentro de 24 horas do início dos sintomas (Figura 1). Os pacientes com idade < 65 anos (n = 474), cirurgia de revascularização do miocárdio (n = 47), sinais de disfunção aguda do ventrículo esquerdo (n = 20), choque cardiogênico (n = 5), edema pulmonar (n = 8), trombose de stent (n = 4), doença infecciosa ou neoplásica aguda ou crônica (n = 6), doença renal crônica moderada a grave (n = 36) e doença hepática crônica (n = 2) foram excluídos deste estudo (n = 602). Durante o acompanhamento, não conseguimos entrar em contato com 37 pacientes. Por fim, concluímos com 119 pacientes elegíveis; 15 pacientes desenvolveram a NIC após a ICP e 104 pacientes constituíram o grupo controle, pareado por idade > 65 anos (Figura 1). A NIC foi caracterizada pelo aumento absoluto de 0,50 mg/dL no nível de creatinina sérica acima da linha de base ou um aumento relativo de ≥ 25% nos níveis de creatinina sérica basal dentro de 48 a 72 horas de exposição ao MC.^14^ Os pacientes do estudo, com idade ≥ 65 anos, foram divididos em dois grupos, o grupo NIC (n = 15) e o grupo sem NIC (n = 104). Para todos os pacientes, o histórico médico, os registros hospitalares, os valores laboratoriais desde a linha de base até o período entre 48 e 72 horas e os achados clínicos foram revisados pelos mesmos dois cardiologistas intervencionistas. Foram identificados fatores de risco cardiovascular, incluindo idade, sexo, diabetes mellitus, hipertensão, hiperlipidemia e tabagismo. Os pacientes com terapia anti-hipertensiva prévia ou pressão arterial de aproximadamente 140/90 mmHg, medida pelo menos duas vezes, foram considerados como hipertensos.^15^ Os pacientes previamente tratados com antidiabético oral e/ou insulinoterapia e os pacientes cuja glicemia em jejum era pelo menos duas vezes maior que 125 mg/dL foram considerados portadores de diabetes mellitus.^16^ A presença de hiperlipidemia foi considerada ao ser obtida uma medida de colesterol total > 200 mg/dL ou colesterol de lipoproteína de baixa densidade > 100 mg/dL ou ainda quando o paciente estava em uso de um medicamento hipolipemiante de acordo com as orientações do Painel de Tratamento de Adultos III.^17^ Os pacientes que usavam tabaco no momento da admissão no serviço de emergência e os que haviam sido ex-fumantes no último mês foram considerados fumantes. O escore de Mehran, que foi relatado por Mehran et al.,^1^ em 2004, inclui hipotensão (5 pontos, se a pressão arterial sistólica for < 80 mmHg durante pelo menos 1 hora, requerendo suporte inotrópico), uso de bomba de balão intra-aórtico (5 pontos), insuficiência cardíaca congestiva (5 pontos, para classe funcional da Nova York Heart Association [NYHA] III/IV ou histórico de edema pulmonar), idade (4 pontos, se > 75 anos), anemia (3 pontos, se hematócrito < 39% para homens e < 36% para mulheres), diabetes mellitus (3 pontos), volume do MC (1 ponto para cada 100 mL) e taxa de filtração glomerular estimada (eTFG) (2 pontos, se a TFG for de 60 a 40, 4 pontos, se a TFG for de 40 a 20, 6 pontos, se a TFG for < 20). Pontuações ≤ 5, 6 a 10, 11 a 15 e > 15 indicam risco de 7,5%, 14%, 26% e 57% para NIC, respectivamente.

Foram coletadas amostras de sangue venoso da veia antecubital imediatamente após a admissão hospitalar, antes da ICP. Foram obtidos o eletrocardiograma de 12 derivações e a pressão arterial no momento da admissão no pronto-socorro. A eTFG de cada paciente foi calculada por meio da equação de Cockcroft-Gault.^18^ O índice de massa corporal foi calculado pela fórmula peso (kg)/ altura^2^ (m^2^). A química sanguínea de rotina, os parâmetros lipídicos e o pico de troponina-I cardíaca foram medidos com um auto-analisador padrão. Foram medidos os hemogramas com um auto-analisador Sysmex K-1000 (Block Scientific, Bohemia, NY, EUA). As amostras foram centrifugadas a 3.000 rpm por 10 minutos e o sobrenadante e o soro foram separados das amostras. Posteriormente, foram congelados a −80 °C até análise posterior. A medição dos níveis de creatinina sérica foi repetida no período entre 48 a 72 horas após a administração do MC.

O diagnóstico de IAMSSST foi feito na presença das seguintes características com base nas definições de diretrizes de prática clínica.^19^ Os pacientes com IAMSSST apresentaram dor ou desconforto torácico típico, ocorrendo em repouso ou esforço mínimo durante pelo menos 10 minutos e o eletrocardiograma inicial mostrou alterações normais ou isquêmicas, como depressões de ST ou inversões da onda T, com nível elevado de troponina I cardíaca, com pelo menos 1 valor acima do limite superior de referência do 99º percentil.

Foram realizados os procedimentos de angiografia coronária por via femoral usando o sistema de angiografia Philips (Optimus 200 DCA e Integris Allura 9, Philips Medical Systems, Eindhoven, Holanda). Um total de 300 mg de ácido acetilsalicílico e uma dose de ataque de clopidogrel (600 mg) e heparina UF (100 mg/kg) foram administrados durante a ICP em todos os pacientes. A angiografia coronária e a ICP foram realizadas usando MC iso-osmolar não iônico (iodixanol, Visipaque 320 mg/100 mL, GE Healthcare, Cork, Irlanda) de acordo com a prática clínica padrão. Foi realizada a ICP da artéria relacionada ao infarto e o volume do MC foi anotado. Pelo menos dois cardiologistas especialistas examinaram a anatomia coronária. Foi utilizado protocolo de hidratação com infusão de 1.000 mL de solução salina isotônica intravenosa (IV) 12 horas antes do procedimento e, após o procedimento, todos os pacientes receberam hidratação IV com solução salina isotônica (1 mL/kg/h) durante pelo menos 12 horas.

Antes da alta hospitalar, cada paciente foi submetido a exame ecocardiográfico transtorácico com um transdutor de 3,5 MHz (Vivid 7 GE Medical System, Horten, Noruega) e a fração de ejeção do ventrículo esquerdo (FEVE) foi calculada por ecocardiografia bidimensional com as medidas do modo M do diâmetro diastólico final e sistólico final do ventrículo esquerdo. As informações de acompanhamento foram obtidas pelos mesmos investigadores dos registros hospitalares; da admissão ao hospital; e de 1, 3, 6 e 12 meses de dados de visita dos pacientes.

Os desfechos desta análise foram derivados de registros hospitalares e atestados de óbito ou comunicação com pacientes e seus familiares por telefone. Eventos cardiovasculares adversos maiores (ECAM) foram definidos como mortalidade por todas as causas, morte cardiovascular, acidente vascular cerebral e re-infarto do miocárdio. Todos os participantes deram consentimento esclarecido por escrito antes da participação e o estudo foi aprovado pelo comitê de ética local (Número: 7/71-04/04/17). Além disso, o estudo foi conduzido de acordo com as disposições da Declaração de Helsinque.

### Análise Estatística

Foram realizadas as análises dos dados utilizando o pacote de software estatístico SPSS versão 22,0 (SPSS Inc., Chicago, IL, EUA). A distribuição normal das variáveis contínuas foi avaliada pelo teste de Kolmogorov-Smirnov. O teste t de amostras independentes ou o teste U de Mann-Whitney foi usado para comparar as variáveis contínuas, dependendo do cumprimento ou não dos pressupostos estatísticos. As variáveis contínuas foram expressas em média e desvio padrão quando normalmente distribuídas, ou a mediana e os percentis 25 e 75 quando não satisfizeram a suposição de normalidade. As variáveis categóricas foram expressas como número (porcentagem). Foi usado o teste de qui-quadrado para comparar as variáveis categóricas. A correlação entre as variáveis foi realizada usando a análise de correlação de ordem de classificação de Spearman. Foi usado o método de Kaplan-Meier para estimar as taxas de sobrevida livre de eventos. Foi realizada análise de regressão logística univariada e as variáveis que se mostraram estatisticamente significativas (p < 0,1) foram analisadas com análise de regressão logística multivariada. Foram calculados a razão de chances e o intervalo de confiança de 95% de cada variável independente. A análise da curva característica de operação do receptor foi realizada para determinar o valor preditivo da KIM-1 o escore de Mehran para a NIC. Foram considerados significativos os valores bicaudais de p < 0,05.

## Resultados

**Figura 2:**
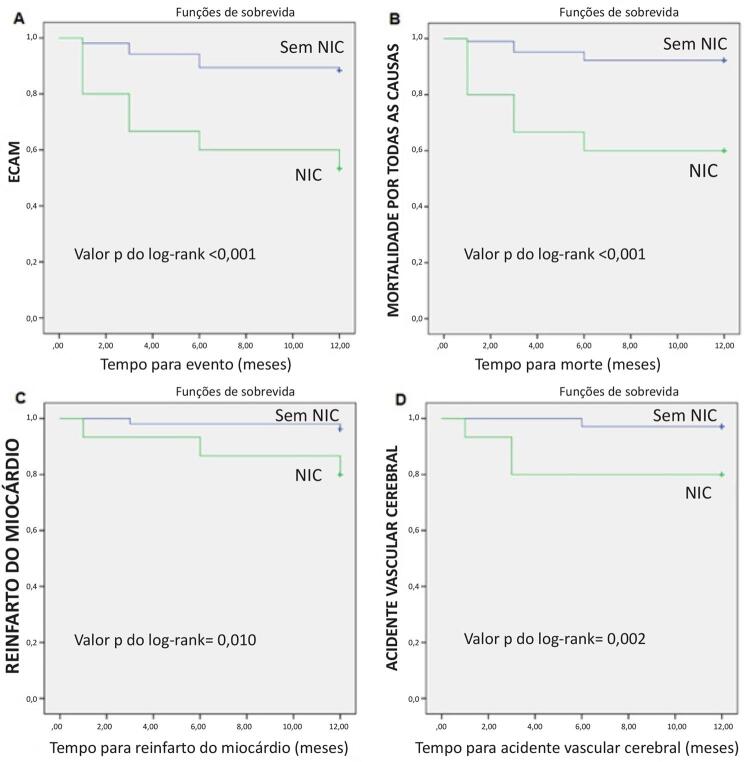
– A) Estimativas de Kaplan-Meier para ECAM. B) Estimativas de Kaplan-Meier para mortalidade por todas as causas. C) Estimativas de Kaplan-Meier para reinfarto do miocárdio. D) Estimativas de Kaplan-Meier para acidente vascular cerebral. ECAM: eventos cardíacos adversos maiores; NIC: nefropatia induzida por contraste.

No presente estudo, inicialmente incluímos 758 pacientes com IAMSSST e concluímos com 119 pacientes elegíveis (79 do sexo masculino; média de idade: 69,96 ± 5,67 anos). No presente estudo, a NIC foi observada em 12,60% (n = 15). Os achados demográficos e laboratoriais são descritos na Tabela 1. Os achados do acompanhamento clínico são descritos na Tabela 2. Hematócrito, FEVE, creatinina, ácido úrico e escore de Mehran foram significativamente associados à eTFG (p < 0,05) (Tabela 3). Não identificamos pacientes com acidente vascular cerebral hemorrágico ou pacientes que necessitassem de diálise durante o seguimento. As estimativas de Kaplan-Meier para ECAM (Figura 2A), mortalidade por todas as causas (Figura 2B), reinfarto do miocárdio (Figura 2C) e taxas de acidente vascular cerebral (Figura 2D) são descritas na Figura 2. A análise de regressão logística multivariada mostrou que o nível de KIM-1 basal (OR = 1,652, IC 95%: 1,20 – 2,27, p = 0,002) e o escore Mehran (OR = 1,457, IC 95%: 1,01 – 2,08, p = 0,039) foram preditores independentes da NIC em pacientes idosos com IAMSSST.

**Tabela 1:** – Características de linha de base e laboratoriais dos pacientes

Variável, n (%)	NIC, n=15 (12,60)	Sem NIC, n=104 (87,40)	Valor p
Idade, anos	70,13±6,68	69,93±5,55	0,613
Sexo masculino, n (%)	13 (86,70)	66 (63,50)	0,075
IMC, kg/m^2^	29,67±4,75	28,66±4,80	0,347
HT, n (%)	12 (80)	65 (62,50)	0,185
DM, n (%)	11 (73,30)	38 (36,50)	0,007
HL, n (%)	11 (73,30)	36 (34,60)	0,004
Tabagismo, n (%)	11 (73,30)	57 (54,80)	0,175
Histórico familiar, n (%)	4 (26,70)	38 (36,50)	0,455
FEVE, %	45±7,07	52,29±7,11	0,001
KIM-1, ng/mL	14,02 (9,53-19,90)	5,41 (3,41-9,03)	<0,001
Glicose, mg/dl	145 (108-252)	113,50 (96-163,75)	0,011
Ácido úrico, mg/dl	8 (6,70-8,70)	5,45 (4,20-6,65)	<0,001
Creatinina, mg/dl	1,20 (0,80-1,50)	0,87 (0,72-1,06)	0,003
eTFG, mL/min	57,79 (43,56-97)	82,85 (67,25-97,87)	0,017
Escore de Mehran	14 (5-22)	5 (2-7)	0,001
HTC, %	37,53±5,49	40,38±4,36	0,017
Plaquetas 10^3^/uL	210 (190-275)	225 (190-267)	0,895
Tempo de interação hospitalar	4,53±1,95	3,11±0,33	<0,001
Triglicerídeos, mg/dL	147 (92-165)	158 (120,25-183,75)	0,247
LDL, mg/dL	113,87±46,42	127,73±31,17	0,135
PA sistólica, mmHg	110 (90-130)	130 (110-140)	0,020
PA diastólica, mmHg	64 (60-70)	70 (65-80)	0,104
Pico de troponina-I, pg/mL	178 (124-5762)	915 (162,75-6171,75)	0,291
CF NYHA	2,33±0,48	2,07±0,46	0,043
EuroSCORE II, %	2,11 (1,60-6,35)	1,58 (1,01-2,65)	0,053
**Medicações**			
IECA, n (%)	6 (40)	62 (59,60)	0,151
BRA, n (%)	6 (40)	34 (32,70)	0,575
Betabloqueadores, n (%)	15 (100)	97 (93,30)	0,300
BCC, n (%)	6 (40)	24 (23,10)	0,158
Estatina, n (%)	14 (93,30)	93 (89,40)	0,638
Nitrato, n (%)	1 (6,70)	44 (42,30)	0,008
AHO, n (%)	10 (66,70)	37 (35,60)	0,021
**Diuréticos, n (%)**	**8 (53,30)**	**37 (35,60)**	**0,185**

*Valores são média ± desvio padrão, números e porcentagens ou a mediana e os percentis 25 e 75. O valor p é para dados categóricos de qui-quadrado. O valor p para o teste t de amostras independentes ou o teste U de Mann-Whitney foi usado para comparar variáveis contínuas. AHO: anti-hiperglicêmicos orais; BCC: bloqueadores dos canais de cálcio; BRA: bloqueadores do receptor da angiotensina; CF NYHA: classe funcional da New York Heart Association; DM: diabetes mellitus tipo 2; eTFG: taxa de filtração glomerular estimada; EuroSCORE: European System for Cardiac Operative Risk Evaluation; FEVE: fração de ejeção do ventrículo esquerdo; HL: hiperlipidemia; HT: hipertensão; HTC: hematócrito; IECA: inibidores da enzima de conversão da angiotensina; IMC: índice de massa corporal; KIM-1: molécula de lesão renal-1; LDL: lipoproteína de baixa densidade; NIC: nefropatia induzida por contraste; PA: pressão arterial.*

**Tabela 2:** – Achados do acompanhamento clínico de um anp

Variável, n (%)	NIC, n=15 (12.60)	Sem NIC, n=104 (87.40)	Valor p
Mortalidade por todas as causas, n (%)	6 (40)	8 (7,70)	<0,001
Morte cardiovascular, n (%)	5 (33,30)	6 (5,80)	0,001
Acidente vascular cerebral, n (%)	3 (20)	3 (2,90)	0,005
Reinfarto do miocárdio, n (%)	3 (20)	4 (3,80)	0,013
**ECAM, n (%)**	**7 (46,70)**	**12 (11,50)**	**0,001**

*Os valores são números e porcentagens. O valor p é para dados categóricos de qui-quadrado. ECAM: eventos cardiovasculares adversos maiores; NIC: nefropatia induzida por contraste.*

**Tabela 3:** – Características de linha de base significativamente associadas à eTFG

Variável	r	Valor p
HTC	0,422	<0,001
FEVE	0,518	<0,001
Creatinina	–0,831	<0,001
Ácido úrico	–0,464	<0,001
**Escore de Mehran**	**–0,664**	**<0,001**

*eTFG: taxa de filtração glomerular estimada; FEVE: fração de ejeção do ventrículo esquerdo; HTC: hematócrito; r: coeficiente de correlação de classificação de Spearman.*

**Figura 3:**
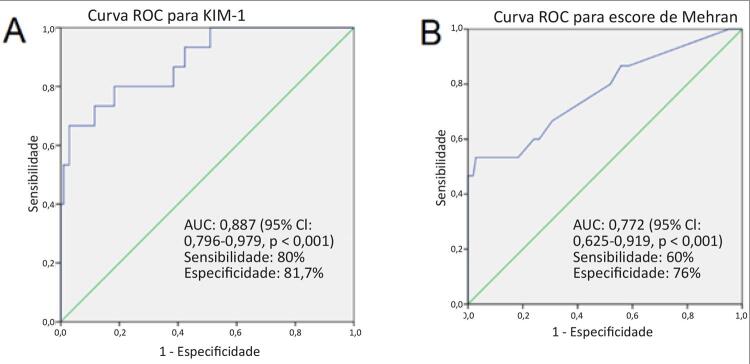
– A) Curva ROC para a especificidade e a sensibilidade da KIM-1. B) Curva ROC para a especificidade e sensibilidade do escore de Mehran. AUC: área sob a curva; IC: intervalo de confiança; KIM-1: molécula de lesão renal-1; ROC: curva característica de operação do receptor.

Na análise da característica de operação do receptor, o nível de KIM-1 acima de 9,49 ng/mL foi preditor da presença de NIC com sensibilidade de 80% e especificidade de 81,70% em pacientes idosos com IAMSSST. A área sob a curva foi 0,887 (IC 95%: 0,796 – 0,979, p < 0,001) (Figura 3A). Além disso, o escore de Mehran acima de 7,5 predisse a presença de NIC com sensibilidade de 60% e especificidade de 76% em pacientes idosos com IAMSSST. A área sob a curva foi 0,772 (IC 95%: 0,625 – 0,919, p = 0,001) (Figura 3B).

## Discussão

O achado principal deste estudo foi que o aumento do nível de KIM-1 e do escore de Mehran foram dois determinantes da NIC em pacientes idosos com IAMSSST. Além disso, em pacientes idosos com IAMSSST, a NIC foi significativamente associada a desfechos desfavoráveis. Demonstramos que valores de KIM-1 acima de 9,49 ng/mL sugerem a presença de NIC em pacientes idosos. O escore de Mehran acima de 7,5 também sugere a presença de NIC em pacientes idosos. Até onde sabemos, este é o primeiro relato na literatura que demonstra a relação entre a NIC e a KIM-1 em pacientes idosos com IAMSSST. Em nosso estudo, os resultados do acompanhamento clínico de um ano mostraram que os ECAM, a mortalidade por todas as causas, o reinfarto do miocárdio e o acidente vascular cerebral foram significativamente maiores no grupo com NIC.

Embora seja controversa a patogênese da NIC em pacientes idosos, as reações oxidativas são geralmente aceitas na patogênese. A NIC é uma doença multifatorial e insuficiência renal basal, insuficiência cardíaca, diabetes mellitus e infarto do miocárdio têm sido propostos para explicar o desenvolvimento da NIC.^20^ Existe um risco aumentado de hospitalização, morbidade e mortalidade em pacientes com a NIC.^21^ Apesar do desenvolvimento de agentes de contraste menos nefrotóxicos, a possibilidade de NIC permanece alta.^22^ A incidência da NIC é > 2% na população geral, mas pode ultrapassar de 20% a 30% em pacientes idosos com diabetes mellitus ou insuficiência cardíaca congestiva.^23^ No presente estudo, a NIC foi observada em 12,60% (n = 15) dos pacientes idosos.

Além disso, Marenzi et al.,^24^ verificaram que níveis menores de FEVE estão associados à NIC.^24^ Kaya et al.,^25^ verificaram que os pacientes que desenvolveram NIC tiveram uma hospitalização acentuadamente prolongada quando comparados ao grupo sem NIC.^25^ Neste estudo consistente com a literatura, verificamos FEVE, eTFG, hematócrito e pressão arterial sistólica significativamente menores no grupo com NIC. Além disso, no presente estudo, verificamos escore de Mehran, nível sérico de KIM-1, glicose, ácido úrico, internação hospitalar prolongada e níveis de creatinina significativamente maiores no grupo com NIC. A internação hospitalar prolongada está associada a um aumento do custo total, que tem importantes implicações clínicas e de saúde. Os médicos precisam estar cientes deste risco potencial.

Adicionalmente, Iakovou et al.,^13^ verificaram que o sexo feminino e a classe funcional da NYHA mais alta são preditores independentes do desenvolvimento da NIC.^13^ Além disso, Zaytseva et al.,^26^ verificaram que pacientes com classificação da NYHA mais elevada apresentam risco aumentado de desenvolver NIC.^26^ Neste estudo consistente com a literatura, verificamos classificações da NYHA mais elevadas no grupo com NIC, mas não verificamos uma correlação entre o sexo e o desenvolvimento da NIC em pacientes idosos com IAMSSST.

Em geral, os túbulos renais proximais expressam níveis muito baixos de KIM-1. No entanto, a expressão da KIM-1 está significativamente aumentada em rins isquêmicos.^27^ Estudos têm sugerido que a KIM-1 interage com a proliferação de células T e de outras proteínas pró-inflamatórias.^7,27^ Macrófagos e linfócitos T são as principais fontes de numerosas citocinas e moléculas que interferem com as células endoteliais, contribuindo para o agravamento das vias inflamatórias. As responsabilidades chaves para as vias fisiopatológicas na lesão tubular são a disfunção endotelial, a inflamação e a produção elevada inexplicada de compostos vasoativos, como endotelina-1 e moléculas de angiotensina.^7,27^A estrutura da proteína da KIM-1 atua como uma molécula de adesão para a superfície celular.^27^ Portanto, especulamos que a KIM-1 pode alterar a adesão celular e modular as interações entre as células epiteliais lesionadas e o conteúdo luminal que inclui cilindros, detritos e células epiteliais viáveis que foram desalojadas do endotélio íntimo dos túbulos renais proximais e podem levar à NIC em pacientes idosos com IAMSSST. A inflamação desempenha um papel importante no estabelecimento e promoção da NIC. Portanto, combinações destes processos pró-inflamatórios parecem plausíveis para esclarecer os mecanismos subjacentes da NIC em pacientes idosos. A KIM-1 não só ajuda na proliferação de macrófagos e linfócitos T, mas também aumenta a produção de citocinas oxidativas.^9^ Os resultados do estudo presente mostram que as concentrações séricas da KIM-1 estão positivamente associadas à NIC em pacientes idosos com IAMSSST. Propomos que a inflamação, a microembolização aterotrombótica e a ativação de neutrófilos e plaquetas, que causam a liberação de radicais livres de oxigênio, enzimas proteolíticas e mediadores pró-inflamatórios que podem causar dano tecidual e endotelial, particularmente em miócitos criticamente lesionados durante o IAMSSST, foram os primeiros mecanismos da NIC em pacientes idosos. Estes mecanismos comuns também atuam em todos os órgãos sensíveis à isquemia, principalmente no coração e nos rins. Assim, podemos usar a KIM-1 como um marcador prognóstico precoce da NIC em pacientes idosos com IAMSSST.

Em relação a este conhecimento, a KIM-1 continua a ser liberada como resultado de danos; também causa danos, por si só, e os rins são vulneráveis a danos diretos. Além disso, verificamos que a KIM-1 é mais sensível e específica do que o escore de Mehran (KIM-1: sensibilidade de 80% e especificidade de 81,70% vs. escore de Mehran: sensibilidade de 60% e especificidade de 76%). Até onde sabemos, este é o primeiro relato na literatura que demonstra a relação entre as concentrações da KIM-1 e a NIC em pacientes idosos com IAMSSST. Nossa hipótese foi a de que, ao medir o nível da KIM-1, seríamos capazes de predizer o risco da NIC em pacientes idosos melhor do que com o escore de Mehran. No entanto, não foi determinado o mecanismo exato da KIM-1 na patogênese da NIC.

Adicionalmente, a NIC é um preditor importante de desfechos cardíacos desfavoráveis em pacientes idosos com IAMSSST.^28^ Shacham et al.,^29^ demonstraram que alguns pacientes mais idosos eram mais propensos a desenvolver a NIC e tinham maior mortalidade por todas as causas, com pior função renal e histórico de insuficiência cardíaca.^29^ Maioli et al.,^30^ verificaram que os pacientes com NIC tiveram uma taxa mais alta de morte em comparação com o grupo sem NIC no acompanhamento de cinco anos.^30^ No presente estudo, os resultados do acompanhamento clínico de um ano demonstraram que os desfechos de ECAM, mortalidade por todas as causas, morte cardiovascular, reinfarto do miocárdio e acidente vascular cerebral foram significativamente maiores no grupo com NIC. Nos pacientes idosos com IAMSSST, encontramos um aumento de 5,2 vezes no risco de mortalidade por todas as causas, de 5,7 vezes no risco de morte cardiovascular, de 6,9 vezes no risco de acidente vascular cerebral, de 5,3 vezes no risco de reinfarto do miocárdio e de 4,1 vezes no risco de ECAM no grupo de pacientes com NIC, em relação ao grupo sem NIC. Com esses resultados, demonstramos que a NIC piora os desfechos de pacientes idosos com IAMSSST.

As estratégias aceitas para a prevenção da NIC são a monitorização do volume de contraste, a redução máxima do uso dos MC e a hidratação dos paciente com solução salina 12 horas antes e após o cateterismo na velocidade de 1 mL/kg/h, de acordo com as diretrizes. A hidratação salina e a expansão do volume podem acelerar a excreção dos MC, diminuir a toxicidade renal direta, diminuir a vasoconstrição e diminuir as espécies reativas de oxigênio.

### Limitações

Primeiramente, a limitação principal do presente estudo é que ele foi realizado com um tamanho relativamente pequeno de amostra. Embora tenha sido realizado um modelo multivariado para ajustar as variáveis de confusão, algum viés era inevitável, visto que se tratava de uma análise de centro único. Ensaios multicêntricos com mais pacientes podem fornecer melhores resultados e mais dados. Segundo, a função renal foi avaliada apenas pelos níveis de creatinina. A medição direta da TFG por meio da coleta de urina de 24 horas é o melhor método para avaliar a função renal, mas é demorada e onerosa para o paciente. Terceiro, para avaliar os resultados clínicos em longo prazo, um período de acompanhamento de um ano pode não ser adequado. Esses constituem fatores limitantes em nosso estudo.

## Conclusão

A concentração sérica da KIM-1 basal e o escore de Mehran são preditores independentes da NIC em pacientes idosos com IAMSSST. Adicionalmente, a mortalidade por todas as causas, morte cardiovascular, reinfarto do miocárdio, acidente vascular cerebral e ECAM foram significativamente maiores em pacientes idosos com IAMSSST no acompanhamento de um ano.
