# Supplementary material for: A Molécula de Lesão Renal-1 está Associada à Nefropatia Induzida por Contraste em Pacientes Idosos com IAMSSST
Source: Arq Bras Cardiol. 2021 Jun 8;116(6):1048–56. [Article in Portuguese] doi: 10.36660/abc.20200172 (PMC8288528; doi:10.36660/abc.20200172)
Supplement: Translation

# Translation: Kidney Injury Molecule-1 Is Associated with Contrast-Induced Nephropathy in Elderly Patients with Non-STEMI

**Authors:** Mustafa Ahmet Huyut

**Affiliations:** 1 Turkey Yeni Yuzyil University, Faculty of Medicine, Department of Cardiology, Istanbul - Turkey

**Keywords:** Kidney Diseases/chemically Induced, Myocardial Infarction SST, Percutaneos Coronary Intervention

## Abstract

**Background:**

Contrast-induced nephropathy (CIN) is associated with an increased risk of major adverse cardiovascular events (MACE), and the association between CIN and oxidative mechanisms is well documented.

**Objective:**

This study aimed to evaluate the relationship between serum levels of kidney injury molecule-1 (KIM-1) and CIN in elderly patients with non-ST-segment elevation myocardial infarction (NSTEMI).

**Methods:**

This study included a total of 758 patients with NSTEMI, who underwent percutaneous coronary intervention (PCI); 15 developed CIN after PCI, and another 104 were the control group, matched for age > 65 years. Baseline to 48-to-72-hour laboratory values and clinical outcomes were recorded. Patients were followed during one year. P values of < 0.05 were considered significant.

**Results:**

CIN was observed in 12.60% of the patients. Serum KIM-1 was significantly higher in the CIN group than in the non-CIN group (14.02 [9.53 – 19.90] vs. 5.41 [3.41 – 9.03], p < 0.001). The Mehran score was significantly higher in the CIN group than in the non-CIN group (14 [5 – 22] vs. 5 [2 – 7], p = 0.001). MACE were significantly higher in the CIN group than in the non-CIN group (7 [46.70%] vs. 12 [11.50%], p = 0.001). Multivariate logistic regression analysis showed that baseline KIM-1 level (OR = 1.652, 95% CI: 1.20 – 2.27, p = 0.002) and Mehran score (OR = 1.457, 95% CI: 1.01 – 2.08, p = 0.039) were independent predictors of CIN in elderly patients with NSTEMI.

**Conclusion:**

Baseline serum KIM-1 concentration and Mehran score are independent predictors of CIN in elderly patients with NSTEMI. Additionally, all-cause mortality, cardiovascular death, myocardial reinfarction, stroke, and MACE were significantly higher in the CIN group at one-year follow-up. (Arq Bras Cardiol. 2021; [online].ahead print, PP.0-0)

## Introduction

Contrast-induced nephropathy (CIN) is associated with increased morbidity, mortality, and increased hospitalizations, due to the application of intravenous or intra-arterial contrast media (CM) during diagnostic or therapeutic vascular procedures.^1^ The incidence of CIN often varies depending on the populations studied and their related comorbidities.^2^ The underlying mechanisms of CIN include endothelial dysfunction, inflammation, vasoconstriction, tubular cell toxicity, free radical injury, reactive oxygen species and oxidative stress, and activation of neutrophils and platelets, which cause the release of oxygen-free radicals, proteolytic enzymes, and proinflammatory mediators that can cause tissue and endothelial damage, particularly in critically injured myocytes.^3,4^ Uric acid, red cell distribution width, the platelet-to-lymphocyte ratio, and the neutrophil-to-lymphocyte ratio have been correlated with CIN in previous studies.^5,6^ Kidney injury molecule-1 (KIM-1) has been related to both the occurrence and severity of acute kidney injury and chronic kidney disease.^7^ KIM-1 is a type-1 transmembrane protein, expressed according to the injury in the proximal tubule of the apical membrane.^8^ Cardiovascular disease has a strong link with acute kidney injury and chronic kidney disease, and cardiovascular events have been reported to be associated with acute kidney injury.^9^ KIM-1 serves as a pro-inflammatory agent with cell-adhesive functions.^7^ In the literature, there are some published studies regarding the relationship between KIM-1^10,11^ and Mehran scores^12,13^ in the development of CIN, but previous studies did not mention which one is the best predictor. Additionally, the previous studies did not make a comparison between KIM-1 and Mehran score for predicting the development of CIN among elderly patients.

We hypothesized that KIM-1 expression is induced in elderly patients with non-ST-segment elevation myocardial infarction (NSTEMI) and is related to CIN due to the pro-inflammatory response and that proximal tubular endothelial damage occurs in this manner. The association between KIM-1 protein levels and CIN in elderly patients with NSTEMI has not yet been addressed in the literature. Understanding which biologic pathways and markers are associated with CIN may allow for the design of future studies to explore the mechanistic link between these pathways and to evaluate the efficacy of interventions designed to reduce the burden of cardiovascular disease and CIN in these patients. For this reason, this study aimed to evaluate the relationship between baseline serum KIM-1 protein levels and CIN in elderly patients with NSTEMI.

## Methods

This study was prospectively conducted between July 2016 and July 2018 at Bezmialem Vakif University Hospital. We enrolled 758 patients who were diagnosed with NSTEMI and who underwent early PCI within 24 hours of the onset of symptoms (Figure 1). Patients with < 65 years (n = 474), coronary artery bypass graft (n = 47), signs of acute left ventricular dysfunction (n = 20), cardiogenic shock (n = 5), pulmonary edema (n = 8), stent thrombosis (n = 4), acute or chronic infective or neoplastic disease (n = 6), moderate to severe chronic kidney disease (n = 36), and chronic liver disease (n = 2) were excluded from this study (n = 602). During the follow-up we could not reach 37 patients. Finally, we concluded with 119 eligible patients; 15 patients developed CIN after PCI, and 104 patients served as the control group, matched for age > 65 years (Figure 1). CIN was characterized as an absolute increase of 0.50 mg/dL in the level of serum creatinine above baseline or ≥ 25% relative increase in the levels of basal serum creatinine within 48 to 72 hours of CM exposure.^14^ The study patients, who were ≥ 65 years old, were divided into two groups, the CIN group (n = 15) and the non-CIN group (n = 104). For all patients, medical history, hospital records, baseline to 48-to-72-hour laboratory values, and clinical findings were reviewed by the same two interventional cardiologists. Cardiovascular risk factors were identified, including age, sex, diabetes mellitus, hypertension, hyperlipidemia, and smoking status. Patients with prior antihypertensive therapy or blood pressure of approximately 140/90 mmHg, measured at least twice, were accepted as having hypertension.^15^ Patients previously treated with oral antidiabetic and/or insulin therapy and patients whose fasting blood glucose was at least twice as high as 125 mg/dL were accepted as having diabetes mellitus.^16^ The presence of hyperlipidemia was considered when a measure of total cholesterol > 200 mg/dL or low-density lipoprotein cholesterol > 100 mg/dL was obtained, or when the patient used a lipid-lowering medication by the provisions of the Adult Treatment Panel III.^17^ Patients who were using tobacco on admission to the emergency service and those who had been ex-smokers in the past month were considered smokers. The Mehran score, which was reported by Mehran et al.^1^ in 2004, includes hypotension (5 points, if systolic blood pressure is < 80 mmHg for at least 1 hour, requiring inotropic support), use of intra-aortic balloon pump (5 points), congestive heart failure (5 points, if New York Heart Association [NYHA] class III/IV or history of pulmonary edema), age (4 points, if > 75 years), anemia (3 points if hematocrit < 39% for men and < 36% for women), diabetes mellitus (3 points), CM volume (1 point per 100 mL), and estimated glomerular filtration rate (eGFR) (2 points if GFR 60 to 40, 4 points if GFR 40 to 20, 6 points if GFR < 20). Scores of ≤ 5, 6 to 10, 11 to 15, and >15 indicate 7.5%, 14%, 26%, and 57% risk for CIN, respectively.

**Figure 1:** – Selection of the study groups.

Venous blood samples from the antecubital vein were taken immediately after admission to the hospital, before PCI. A 12-lead electrocardiogram and blood pressure were obtained at the time of admission to the emergency department. The eGFR of each patient was calculated using the Cockcroft-Gault equation.^18^ Body mass index was calculated with the formula weight (kg)/ height^2^ (m^2^). Routine blood chemistry, lipid parameters, and peak cardiac troponin-I were measured with a standard auto-analyzer. Blood counts were measured with a Sysmex K-1000 (Block Scientific, Bohemia, NY, USA) auto-analyzer. Samples were centrifuged at 3000 rpm for 10 minutes, and the supernatant and serum were separated from the samples. Subsequently, they were frozen at −80 °C until further analysis. Serum creatinine levels measurement was repeated at 48 to 72 hours after CM administration.

The diagnosis of NSTEMI was made in the presence of the following characteristics based on definitions from clinical practice guidelines.^19^ NSTEMI patients had typical chest pain or discomfort occurring at rest or minimal exertion for at least 10 minutes and the initial electrocardiogram showed normal or ischemic changes such as ST depressions or T-wave inversions, with elevated cardiac troponin-I level, with at least 1 value above the 99th percentile upper reference limit.

Coronary angiography procedures were conducted via the femoral approach using the Philips (Optimus 200 DCA and Integris Allura 9, Philips Medical Systems, Eindhoven, Netherlands) angiography system. A total of 300 mg acetylsalicylic acid and a loading dose of clopidogrel (600 mg) and UF heparin (100 mg/kg) were administered during PCI in all patients. Coronary angiography and PCI were conducted using nonionic, iso-osmolar CM (iodixanol, Visipaque 320 mg/100 mL, GE Healthcare, Cork, Ireland) according to standard clinical practice. PCI of the infarct-related artery was performed and CM volume was noted. At least two expert cardiologists examined coronary anatomy. A hydration protocol was used with 1,000 mL of intravenous (IV) isotonic saline infusion starting 12 hours before the procedure, and after the procedure, all patients received IV hydration with isotonic saline (1 mL/kg/h) for at least 12 hours.

Before discharge, each patient underwent transthoracic echocardiographic examination with a 3.5-MHz transducer (Vivid 7 GE Medical System, Horten, Norway), and left ventricular ejection fraction (LVEF) was calculated by two-dimensional echocardiography with the M-mode measurements of left ventricular end-diastolic and end-systolic diameters. Follow-up information was obtained from hospital records; admission to the hospital; and 1, 3, 6, and 12 months of patient visit data, by the same investigators.

The endpoints of this analysis were derived through hospital records and death certificates, or communication with patient families by telephone. Major adverse cardiovascular events (MACE) were defined as all-cause mortality, cardiovascular death, stroke, and myocardial re-infarction. All participants gave written informed consent before participation, and the study was approved by the local ethics committee (Number:7/71-04/04/17). Furthermore, the study was conducted in accordance with the provisions of the Helsinki Declaration.

### Statistical Analysis

Data analyses were performed using SPSS version 22.0 statistical software package (SPSS Inc., Chicago, IL, USA). The normal distribution of a continuous variable was assessed using the Kolmogorov-Smirnov test. The independent samples t test or the Mann-Whitney U test was used to compare continuous variables depending on whether statistical assumptions were fulfilled or not. Continuous variables were expressed as mean and standard deviation if normally distributed, or median and 25th and 75th percentiles if they did not satisfy the normal assumption. Categorical variables were expressed as number (percentage). The chi-square test was used to compare categorical variables. The correlation between variables was performed using Spearman’s rank-order correlation analysis. The Kaplan-Meier method was used to estimate event-free survival rates. Univariate logistic regression analysis was performed, and the variables that were found to be statistically significant (p < 0.1) were analyzed with multivariate logistic regression analysis. The odds ratio and 95% confidence interval of each independent variable were calculated. Receiver operating characteristic curve analysis was performed to determine the predictive value of KIM-1 and Mehran score for CIN. Two-tailed p values of < 0.05 were considered significant.

## Results

In this study, we initially included 758 patients with NSTEMI, and we concluded with 119 eligible patients (79 male; mean age: 69.96 ± 5.67 years). In this study, CIN was observed in 12.60% (n = 15). Demographic and laboratory findings are described in Table 1. Clinical follow-up findings are described in Table 2. Hematocrit, LVEF, creatinine, uric acid, and Mehran score were significantly associated with eGFR (p < 0.05) (Table 3). We did not identify any patients with hemorrhagic stroke or patients requiring dialysis during follow-up. Kaplan-Meier estimates for MACE (Figure 2A), all-cause mortality (Figure 2B), myocardial reinfarction (Figure 2C), and stroke rates (Figure 2D) are described in Figure 2. Multivariate logistic regression analysis showed that baseline KIM-1 level (OR = 1.652, 95% CI: 1.20 – 2.27, p = 0.002) and Mehran score (OR = 1.457, 95% CI: 1.01 – 2.08, p = 0.039) were independent predictors of CIN in elderly patients with NSTEMI.

**Table 1:** – Baseline and laboratory characteristics of the patients

Variable, n (%)	CIN, n=15 (12.60)	Non-CIN, n=104 (87.40)	p value
Age, years	70.13±6.68	69.93±5.55	0.613
Male sex, n (%)	13 (86.70)	66 (63.50)	0.075
BMI, kg/m^2^	29.67±4.75	28.66±4.80	0.347
HT, n (%)	12 (80)	65 (62.50)	0.185
DM, n (%)	11 (73.30)	38 (36.50)	0.007
HL, n (%)	11 (73.30)	36 (34.60)	0.004
Smoking, n (%)	11 (73.30)	57 (54.80)	0.175
Family history, n (%)	4 (26.70)	38 (36.50)	0.455
LVEF, %	45±7.07	52.29±7.11	0.001
KIM-1, ng/mL	14.02 (9.53-19.90)	5.41 (3.41-9.03)	<0.001
Glucose, mg/dl	145 (108-252)	113.50 (96-163.75)	0.011
Uric acid, mg/dl	8 (6.70-8.70)	5.45 (4.20-6.65)	<0.001
Creatinine, mg/dl	1.20 (0.80-1.50)	0.87 (0.72-1.06)	0.003
eGFR, mL/min	57.79 (43.56-97)	82.85 (67.25-97.87)	0.017
Mehran score	14 (5-22)	5 (2-7)	0.001
HTC, %	37.53±5.49	40.38±4.36	0.017
Platelets 10^3^/uL	210 (190-275)	225 (190-267)	0.895
Length of hospital stay, days	4.53±1.95	3.11±0.33	<0.001
Triglycerides, mg/dL	147 (92-165)	158 (120.25-183.75)	0.247
LDL, mg/dL	113.87±46.42	127.73±31.17	0.135
Systolic BP, mmHg	110 (90-130)	130 (110-140)	0.020
Diastolic BP, mmHg	64 (60-70)	70 (65-80)	0.104
Peak troponin-I, pg/mL	178 (124-5762)	915 (162.75-6171.75)	0.291
NYHA FC	2.33±0.48	2.07±0.46	0.043
EuroSCORE II, %	2.11 (1.60-6.35)	1.58 (1.01-2.65)	0.053
**Medications**			
ACEI, n (%)	6 (40)	62 (59.60)	0.151
ARB, n (%)	6 (40)	34 (32.70)	0.575
Beta blockers, n (%)	15 (100)	97 (93.30)	0.300
CCB, n (%)	6 (40)	24 (23.10)	0.158
Statin, n (%)	14 (93.30)	93 (89.40)	0.638
Nitrate, n (%)	1 (6.70)	44 (42.30)	0.008
OAD, n (%)	10 (66.70)	37 (35.60)	0.021
**Diuretic, n (%)**	**8 (53.30)**	**37 (35.60)**	**0.185**

*Values are mean ± SD, numbers and percentages or median and 25th and 75th percentiles. The p value is for categorical data from Chi-square. The p value for independent samples t test or the Mann-Whitney U test was used to compare continuous variables. ACEI: angiotensin-converting enzyme inhibitors; ARB: angiotensin receptor blockers; BMI: body mass index; BP: blood pressure; CCB: calcium channel blockers; CIN: contrast-induced nephropathy; DM: diabetes mellitus type 2; eGFR: estimated glomerular filtration rate; EuroSCORE: European System for Cardiac Operative Risk Evaluation; HL: hyperlipidemia; HT hypertension; HTC: hematocrit; KIM-1: Kidney injury molecule-1; LVEF: left ventricular ejection fraction; LDL: low-density lipoprotein; NYHA FC: New York Heart Association functional class; OAD: oral antihyperglycemic drugs.*

**Table 2:** – One-year clinical follow-up findings

Variable, n (%)	CIN, n=15 (12.60)	Non-CIN, n=104 (87.40)	p value
All-cause mortality, n (%)	6 (40)	8 (7.70)	<0.001
Cardiovascular death, n (%)	5 (33.30)	6 (5.80)	0.001
Stroke, n (%)	3 (20)	3 (2.90)	0.005
Myocardial reinfarction, n (%)	3 (20)	4 (3.80)	0.013
**MACE, n (%)**	**7 (46.70)**	**12 (11.50)**	**0.001**

*Values are numbers and percentages. The p value is for categorical data from chi-square. CIN: contrast-induced nephropathy; MACE: major adverse cardiovascular events.*

**Table 3:** – Baseline characteristics significantly associated with eGFR

Variable	r	p value
HTC	0.422	<0.001
LVEF	0.518	<0.001
Creatinine	–0.831	<0.001
Uric acid	–0.464	<0.001
**Mehran score**	**–0.664**	**<0.001**

*eGFR: estimated glomerular filtration rate; HTC: hematocrit; LVEF: left ventricular ejection fraction; r: Spearman’s rank correlation coefficient.*

**Figure 2:** – A) Kaplan-Meier estimates for MACE. B) Kaplan-Meier estimates for all-cause mortality. C) Kaplan-Meier estimates for myocardial reinfarction. D) Kaplan-Meier estimates for stroke. CIN: contrast-induced nephropathy; MACE: major adverse cardiac events.

In receiver operating characteristic analysis, KIM-1 above 9.49 ng/mL predicted the presence of CIN with 80% sensitivity and 81.70% specificity in elderly patients with NSTEMI. The area under the curve was 0.887 (95% CI: 0.796 – 0.979, p < 0.001) (Figure 3A). Moreover, Mehran score above 7.5 predicted the presence of CIN with 60% sensitivity and 76% specificity in elderly patients with NSTEMI. The area under the curve was 0.772 (95% CI: 0.625 – 0.919, p = 0.001) (Figure 3B).

**Figure 3:** – A) ROC curve for the specificity and sensitivity of KIM-1. B) ROC curve for the specificity and sensitivity of the Mehran score. AUC: area under the curve; CI: confidence interval; KIM-1: kidney injury molecule-1; ROC: receiver operating characteristic curve.

## Discussion

The key finding of this study was that increased KIM-1 level and Mehran score were two determinants of CIN in elderly patients with NSTEMI. Additionally, in elderly patients with NSTEMI, CIN was significantly associated with poor outcomes. We have shown that values of KIM-1 above 9.49 ng/mL suggest the presence of CIN in elderly patients. Moreover, Mehran score above 7.5 suggests the presence of CIN in elderly patients. To the best of our knowledge, this is the first report in the literature demonstrating the relationship between CIN and KIM-1 in elderly patients with NSTEMI. In our study, the results of one-year clinical follow-up showed that MACE, all-cause mortality, myocardial reinfarction, and stroke were significantly higher in the CIN group.

Although the pathogenesis of CIN is controversial in elderly patients, oxidative reactions are generally accepted in the pathogenesis. CIN is a multifactorial disease and baseline renal insufficiency, heart failure, diabetes mellitus, and myocardial infarction have been proposed to explain the development of CIN.^20^ There is an increased risk of hospitalization, morbidity, and mortality rates in patients with CIN.^21^ Despite the development of less nephrotoxic contrast agents, the possibility of CIN remains high.^22^ The incidence of CIN is > 2% in the general population, but it can exceed 20% to 30% in elderly patients with diabetes mellitus or congestive heart failure.^23^ In this study, CIN was observed in 12.60% (n = 15) of elderly patients.

Moreover, Marenzi et al.^24^ found that lower LVEF is associated with CIN.^24^ Kaya et al.^25^ found that patients who developed CIN had a markedly extended hospitalization when compared with the non-CIN group.^25^ In this study consistent with the literature, we have seen significantly lower LVEF, eGFR, hematocrit, and systolic BP in the CIN group. Moreover in this study, we have seen significantly higher Mehran score, serum KIM-1 level, glucose, uric acid, extended hospitalization, and creatinine level in the CIN group. Extended hospitalization is associated with an increased total cost, which has important clinical and health care implications. Physicians need to be aware of this potential risk.

Additionally, Iakovou et al.^13^ found that the female sex and higher NYHA score are independent predictors of CIN development.^13^ Also, Zaytseva et al.^26^ found that patients who have higher NYHA scores have increased risk of developing CIN.^26^ In this study consistent with the literature, we have found higher NYHA scores in the CIN group, but we did not find any correlation between sex and the development of CIN in elderly patients with NSTEMI.

In general, proximal renal tubules express very low KIM-1 levels. However, KIM-1 expression is significantly increased in ischemic kidneys.^27^ Studies have suggested that KIM-1 interacts with the proliferation of T cells and other pro-inflammatory proteins.^7,27^ Macrophages and T lymphocytes are the main sources of numerous cytokines and molecules interfering with endothelial cells, contributing to an aggravation of inflammatory pathways. The key responsibilities for the pathophysiological pathways on tubular injury are endothelial dysfunction, inflammation, and unexplained elevated production of vasoactive compounds, such as endothelin-1 and angiotensin molecules.^7,27^The protein structure of KIM-1 acts as an adhesion molecule for the cell surface.^27^ Therefore, we speculate that KIM-1 might alter cellular adhesion and modulate interactions between injured epithelial cells and the luminal contents that include casts, debris, and viable epithelial cells that have become dislodged from the intimal endothelium of the proximal renal tubules and might lead to CIN in elderly patients with NSTEMI. Inflammation plays an important role in establishing and promoting CIN. Therefore, combinations of these pro-inflammatory processes appear plausible to clarify the underlying mechanisms of CIN in elderly patients. KIM-1 not only helps in the proliferation of macrophages and T lymphocytes, but also enhances the production of oxidative cytokines.^9^ The results of this study show that serum KIM-1 concentrations are positively associated with CIN in elderly patients with NSTEMI. We propose that inflammation, atherothrombotic microembolization, and activation of neutrophils and platelets, which cause the release of oxygen-free radicals, proteolytic enzymes, and proinflammatory mediators that can cause tissue and endothelial damage, particularly in critically injured myocytes during NSTEMI, were the initial mechanisms of CIN in elderly patients. These common mechanisms also work on every ischemic-sensitive organ, particularly on the heart and kidneys. Thus, we can use KIM-1 as an early prognostic marker of CIN in elderly patients with NSTEMI.

Regarding this knowledge, KIM-1 continues to be released as a result of damage; it also causing damage, in itself, and the kidneys are vulnerable to direct damage. Moreover, we have found that KIM-1 is more sensitive and specific than the Mehran score (KIM-1: 80% sensitivity and 81.70% specificity vs. Mehran score: 60% sensitivity and 76% specificity). To the best of our knowledge, this is the first report in the literature demonstrating the relationship between KIM-1 concentrations and CIN in elderly patients with NSTEMI. We hypothesized that, by measuring the KIM-1 level, we would be able to predict the risk of CIN in elderly patients better than the with the Mehran score. However, the exact mechanism of KIM-1 in the pathogenesis of CIN has not been determined.

Also, CIN is an important predictor of poor cardiac outcomes in elderly patients with NSTEMI.^28^ Shacham et al.^29^ demonstrated that some older patients were more likely to develop CIN and have higher all-cause mortality, with worse renal function, and history of heart failure.^29^ Maioli et al.^30^ found that patients with CIN had a higher rate of death compared to the non-CIN group at five years of follow-up.^30^ In this study, one-year clinical follow-up findings demonstrated that MACE, all-cause mortality, cardiovascular death, myocardial reinfarction, and stroke outcomes were significantly higher in the CIN group. In elderly patients with NSTEMI, we have found a 5.2-fold increase in the risk of all-cause mortality, a 5.7-fold in the risk of cardiovascular death, a 6.9-fold in the risk of stroke, a 5.3-fold in the risk of myocardial reinfarction, and a 4.1-fold in the risk of MACE in the CIN group with respect to the group of patients without CIN. With these results, we have shown that CIN worsens the outcomes of elderly patients with NSTEMI.

The accepted strategies for preventing CIN are monitoring the contrast volume, reducing the use of CM as much as possible, and hydrating the patient with saline solution 12 hours before and after catheterization at a speed of 1 mL/kg/h, according to the guidelines. Saline hydration and volume expansion could speed up CM excretion, decrease direct renal toxicity, decrease vasoconstriction, and decrease reactive oxygen species.

### Limitations

First, the main limitation of the present study is that it was conducted with a fairly small sample size. Although a multivariate model was conducted to adjust confounding variables, a bias was inevitable, given that this was a single-center analysis. Multicenter trials with more patients could provide better results and more data. Second, renal function was only assessed by creatinine levels. Direct measurement of GFR through 24-hour urine collection is the best method for assessing kidney function, but it is time-consuming and onerous for the patient. Third, to assess long-term clinical results, a follow-up period of one year may not be adequate. These are limiting factors in our study.

## Conclusion

Baseline serum KIM-1 concentration and Mehran score are independent predictors of CIN in elderly patients with NSTEMI. Additionally, all-cause mortality, cardiovascular death, myocardial reinfarction, stroke, and MACE were significantly higher in the CIN group at one-year follow-up.
